# Molecular Dynamics Simulations Reveal the Mechanisms of Allosteric Activation of Hsp90 by Designed Ligands

**DOI:** 10.1038/srep23830

**Published:** 2016-04-01

**Authors:** Gerolamo Vettoretti, Elisabetta Moroni, Sara Sattin, Jiahui Tao, David A. Agard, Anna Bernardi, Giorgio Colombo

**Affiliations:** 1 Istituto di Chimica del Riconoscimento Molecolare, CNR (ICRM-CNR), via Mario Bianco, 9, 20131, Milan, Italy; 2 Università degli Studi di Milano, Dipartimento di Chimica, via Golgi, 19, 20133, Milan, Italy; 3 Howard Hughes Medical Institute and Dept. of Biochemistry & Biophysics, University of California, San Francisco, 94158 USA

## Abstract

Controlling biochemical pathways through chemically designed modulators may provide novel opportunities to develop therapeutic drugs and chemical tools. The underlying challenge is to design new molecular entities able to act as allosteric chemical switches that selectively turn on/off functions by modulating the conformational dynamics of their target protein. We examine the origins of the stimulation of ATPase and closure kinetics in the molecular chaperone Hsp90 by allosteric modulators through atomistic molecular dynamics (MD) simulations and analysis of protein-ligand interactions. In particular, we focus on the cross-talk between allosteric ligands and protein conformations and its effect on the dynamic properties of the chaperone’s active state. We examine the impact of different allosteric modulators on the stability, structural and internal dynamics properties of Hsp90 closed state. A critical aspect of this study is the development of a quantitative model that correlates Hsp90 activation to the presence of a certain compound, making use of information on the dynamic adaptation of protein conformations to the presence of the ligand, which allows to capture conformational states relevant in the activation process. We discuss the implications of considering the conformational dialogue between allosteric ligands and protein conformations for the design of new functional modulators.

Protein functions are determined by their internal dynamics and are fine-tuned by the interactions with effectors of different chemical origins^1^,^2^,^3^. Most chemical interventions on proteins and enzymes focus on the identification of small molecule inhibitors designed to abrogate their activities^4^, or on the development of agonists that induce active-like conformations of the target and subsequent signaling responses by binding to known orthosteric sites^5^.

In contrast, the rational search for allosteric activators of protein functions is still relatively unexplored^6^,^7^. However, allosteric activators may provide new opportunities to develop chemical tools to probe the role of protein activity in cellular phenotypes: indeed new information can be gained by turning on a signaling pathway starting from a specific node of the underlying protein network^7^,^8^. Allosteric activators can in principle modulate receptor function while still allowing the possibility of orthosteric agonist or antagonist binding. In this context, allosteric ligands can act as affinity modulators by changing the affinity of the orthosteric ligand for the receptor, as well as induce conformational changes that in turn modulate the efficacy of the orthosteric ligand in determining cellular responses.

Moreover, activators can help identify and characterize allosteric sites and mechanisms for the discovery of novel drug candidates: indeed, since allostery represents one of the most relevant means to regulate protein function, drug discovery can be extended to target regulatory protein pockets. It has previously been shown that sequence variation in allosteric pockets could aid the design of highly specific drugs that would bind only specific members of a protein family. Finally, allosteric ligands can potentially modulate the balance between the target protein conformations that are presented to other interacting proteins, generating the possibility to perturb protein-protein interactions (PPIs) through low molecular weight ligands^9^,^10^.

Defining the principles of ligand recognition and allostery is thus important for understanding the molecular links between small-molecule binding and observed effects. This would in turn facilitate the generation of novel chemical tools and therapeutic agents. Experimental approaches based on X-ray crystallography, tethering, enzymatic, biochemical and phenotypical assays have been used to characterize the activation of enzymatic functions determined by small molecules^6^,^7^,^9^,^10^. Despite these exciting advances, experimental techniques are still limited in their potential to provide insight at an atomic level into the allosteric activation process itself. To understand activation mechanisms at atomistic detail, we can turn to theoretical and computational approaches^10^,^11^. The latter methods can help address open questions such as the role of variations in protein structures and internal dynamics incurred by binding at an allosteric site, and the possibility to quantitatively correlate ligand binding, dynamic-changes and observed modulation of a certain activity^12^.

In this paper, we will address the problem of rationalizing the observed allosteric activation of the 90 kDa Heat Shock Protein (Hsp90) molecular chaperone through a set of newly designed ligands^12^. Members of the Hsp90 family are hub proteins that control the crossroads of fundamental pathways required for cell development and maintenance. The disregulation of Hsp90 functions, often associated with its overexpression, has been shown to be at the basis of disease states, such as cancer and neurodegeneration^13^,^14^,^15^,^16^,^17^,^18^,^19^. At the molecular level, the activities of Hsp90 are regulated by ATP binding and hydrolysis: the type of bound nucleotide selects the conformational sub-states and the transitions among them, which ultimately determine the chaperone’s functional properties^20^.

From a structural point of view, Hsp90 is a homodimer (see schematic representation in Fig. 1), whereby each protomer consists of three distinct structural domains: an N-terminal regulatory Domain (NTD), where the ATP binding site is located, a Middle Domain (M-domain) composed of a Large (LMD) and a Small (SMD) subdomain, which completes the ATPase site necessary for ATP hydrolysis and binds client proteins, and a C-terminal Domain (CTD) that is involved in dimerization^21^,^22^,^23^,^24^,^25^. A bipartite client binding site made of residues from both the MD and CTD has been identified^26^,^27^. This structural organization is common to the various Hsp90 homologues from different organisms and cellular compartments^21^,^22^,^23^,^24^,^25^,^28^.

Although the exact mechanisms of coupling between ATP-binding/hydrolysis and the chaperone cycle remain partly elusive, combining available crystal structure information and solution state data, Agard and collaborators have developed a general model that recapitulates the main traits of nucleotide dependent conformational changes in Hsp90. In particular, ATP binding in both NTDs from the two protomers stabilizes a closed NTD-dimerized activated state, characterized by an excess structural strain that is ultimately responsible for the population of an asymmetric conformation^28^,^29^. Hydrolysis of one of the two ATPs could then cause a change in symmetry (either to a symmetric state or possibly an opposite asymmetric state), providing a mechanism to directly couple hydrolysis to rearrangement of the client binding residues identified in the MD:CTD region^21^,^22^,^23^,^28^. A simple schematics of the mechanism is reported in Fig. 1A.

With the aim of shedding light on the roles of the ATPase in regulating the conformational and cell-functional properties of the chaperone, we recently designed a library of small molecules that *activate* Hsp90 ATPase by targeting an allosteric site located 65 Å away from the active site^12^. The latter, located at the MD:CTD border in a region largely overlapping with the client binding site at the interface between the two protomers, was identified through the characterization of long-range dynamic coordination patterns based on the analysis of residue-pair distance fluctuations^30^,^31^,^32^. The characterization of the stereochemical and dynamic properties of the allosteric site constituted the basis for the optimization of the allosteric ligands, generating compounds able to accelerate the chaperone enzymatic activity up to 6 times.

Herein, we examine the origin of the allosteric activation of Hsp90 and set out to lay the basis for the development of a predictive model of ligand-induced activation of the protein’s functional dynamics. Through the use of comparative analyses of molecular dynamics (MD) simulations of different complexes of the chaperone in the presence of allosteric activators, and known inhibitors as controls, we provide molecular-level insights into how ligand binding at an allosteric site can affect protein structure, dynamics and, consequently, enzymatic activity. We also provide a first, dynamics-based, quantitative model that links the structure of the allosteric ligands to experimentally measured activation effects. Finally, we discuss the implications of our work for activator design.

## Results and Discussion

**Figure 1:**
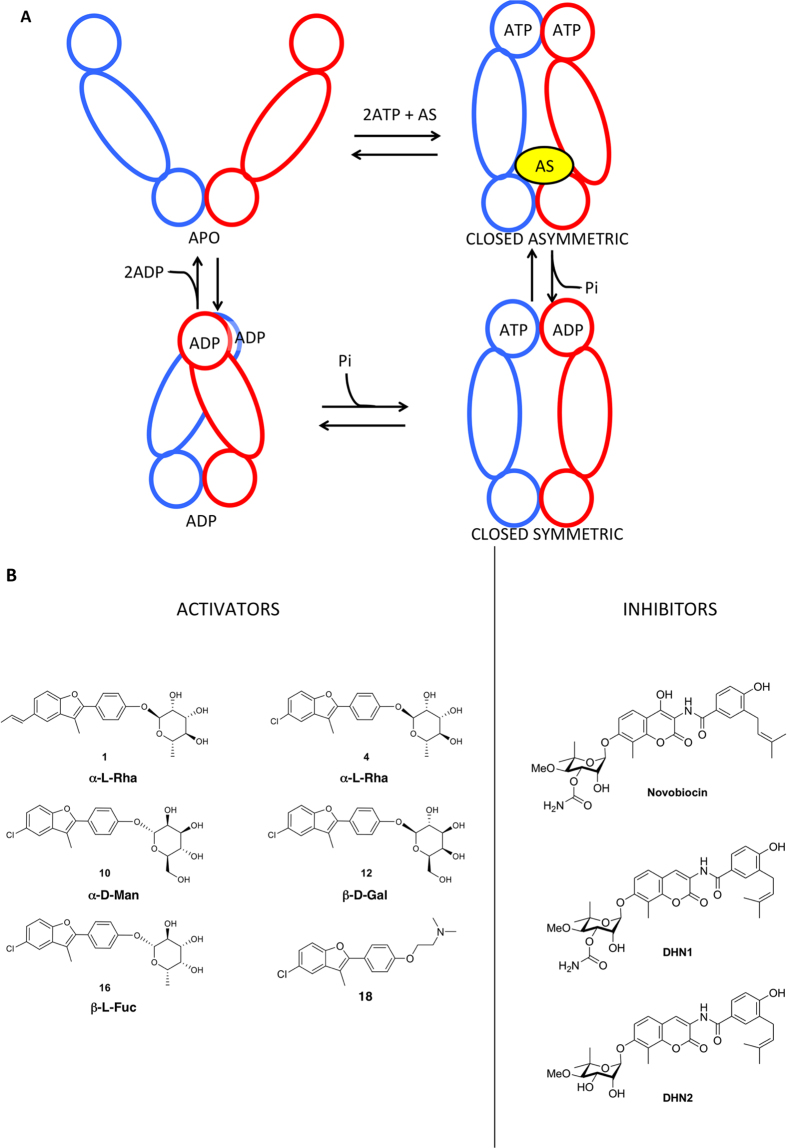
Hsp90 conformational cycle and structures of allosteric ligands. (**A**) In the apo state, Hsp90 populates a number of states with open conformations. Upon ATP and Allosteric Stimulator (AS) binding, the chaperone shifts to an asymmetric closed conformation with significant strain, with particular distortions at the MD:CTD interface. After the first ATP is hydrolyzed, strain is relieved, and the chaperone moves through symmetric states, returning to the open conformation. (**B**) Molecular structures of the activators and inhibitors studied in this work.

To start building a structural and dynamic model of the mechanisms of Hsp90 activation induced by the allosteric ligands, we carried out MD simulations of closed ATP-bound Hsp90 in complex with compounds **1, 4, 10, 12, 16, 18** (Fig. 1B) at the allosteric site (Fig. 1A, top-right), and compared the results with those obtained in the absence of compounds (ATP-only). The structures, ATPase and closure rate stimulation effects for the compounds are reported in Table 1. We focused on the closed conformation of Hsp90 as a model of the activated state of the protein. In this context, our aim is not full sampling of conformational changes or the exhaustive exploration of the highly complex free energy landscape of the protein and its modulation by the ligands. Our goal is to shed light on the microscopic details of Hsp90 internal dynamics induced by specific ligands that can ultimately be linked to the activation of a specific functional state, namely the one related to ATPase activation. This is expected to determine dynamic signatures for defined ligand states of the chaperone, providing information on allosteric mechanisms.

### Structural analysis: Allosteric Activators Stabilize Closed Compact States of the Dimer

**Figure 2:**
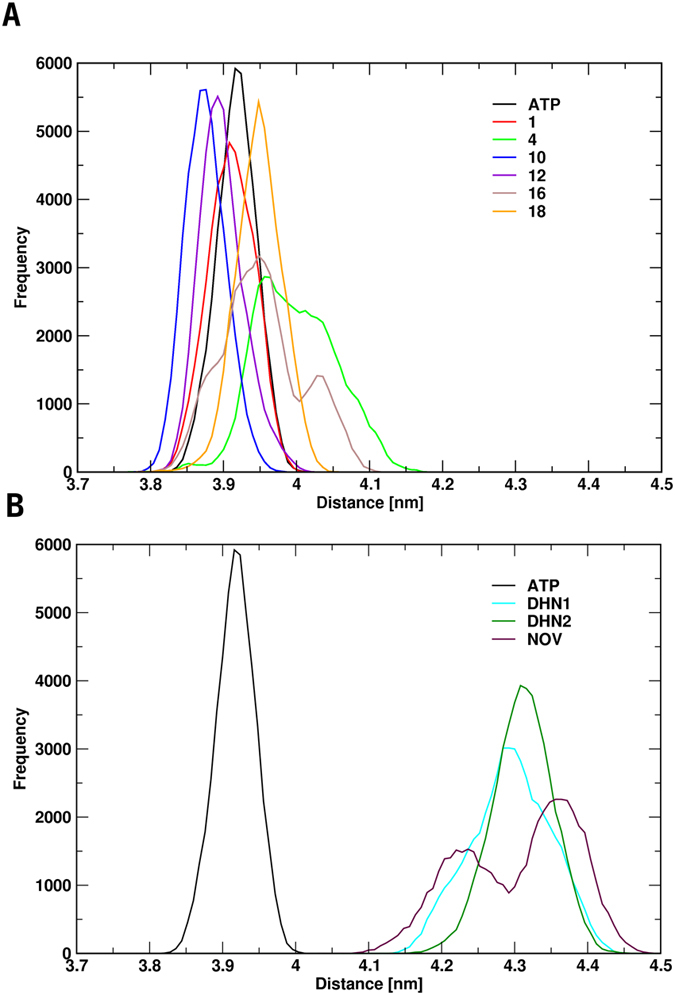
Distribution of the distance between the centers of mass of the N-terminal domains in the Hsp90 complexes with different ligands. Panels **A** and **B** show the distributions of the distances of the N-terminal domains of the complexes formed with the activating ligands (**A**) and the inhibiting ligands (**B**). As a reference, all panels include the distributions of the interprotomer distances of the simulations in the presence of ATP only (APO).

We first analyzed the frequency distribution of distances between the centers of mass of the N-terminal domains of the two protomers in the presence of a certain allosteric ligand, compared to the ATP-only bound states. Only backbone atoms were considered in calculation. This parameter informs on the propensity of Hsp90 to populate the closed, N-terminal dimerized state in the presence of the allosteric ligand. For the complexes of Hsp90 with ATP and allosteric *activators*, the peaks of the frequency distributions are at similar or slightly shorter distances than the ATP-only simulation, indicating an enhanced propensity to populate closed conformations (Fig. 2A). This latter is considered to represent the activated state of the chaperone^31^,^32^.

In contrast, control simulations run with known *inhibitors* (Novobiocin, DHN1, DHN2) show that the peaks for the *inhibitors* are consistently centered at larger NTD-NTD distances (Fig. 2B), indicating a propensity for stabilizing a more open, inactive state^31^,^32^.

Overall, within the limitations of MD sampling, these results indicate that the allosteric activators tend to maintain the protein in an ensemble of structures similar to the ones observed for the ATP-only case, where the N-terminal organization remains tightly closed. The population of the latter, initiated by ATP (or ATP analogs), is a hallmark of Hsp90 activation and represents the catalytically active state of the protein that allows ATPase activity. In contrast, inhibitors appear to favor less compact conformations by shifting the peak distributions by about 0.5 nanometers (5 Å) towards more open structures, which may be associated to the selection of non-reactive states^31^,^32^. As a caveat, it must be stated that these considerations clearly have a mostly qualitative value and more efficient sampling methods, based on enhanced sampling techniques, coupled to longer simulation timescales might be needed to quantitatively profile the effects of allosteric ligands on Hsp90 free energy landscape.

### Allosteric Activators Distort Hsp90 Inducing an Asymmetric State

We next analyzed the large-scale distortions determined by the allosteric ligands. To do this, we evaluated the time evolution of the angle formed by the principal axis of the N-M domains of each protomer in simulated snapshots with the principal axis of the same N-M domain in the 2CG9.pdb closed structure, derived from yeast and used a reference, after alignment of the structures on the CTDs.

**Figure 3:**
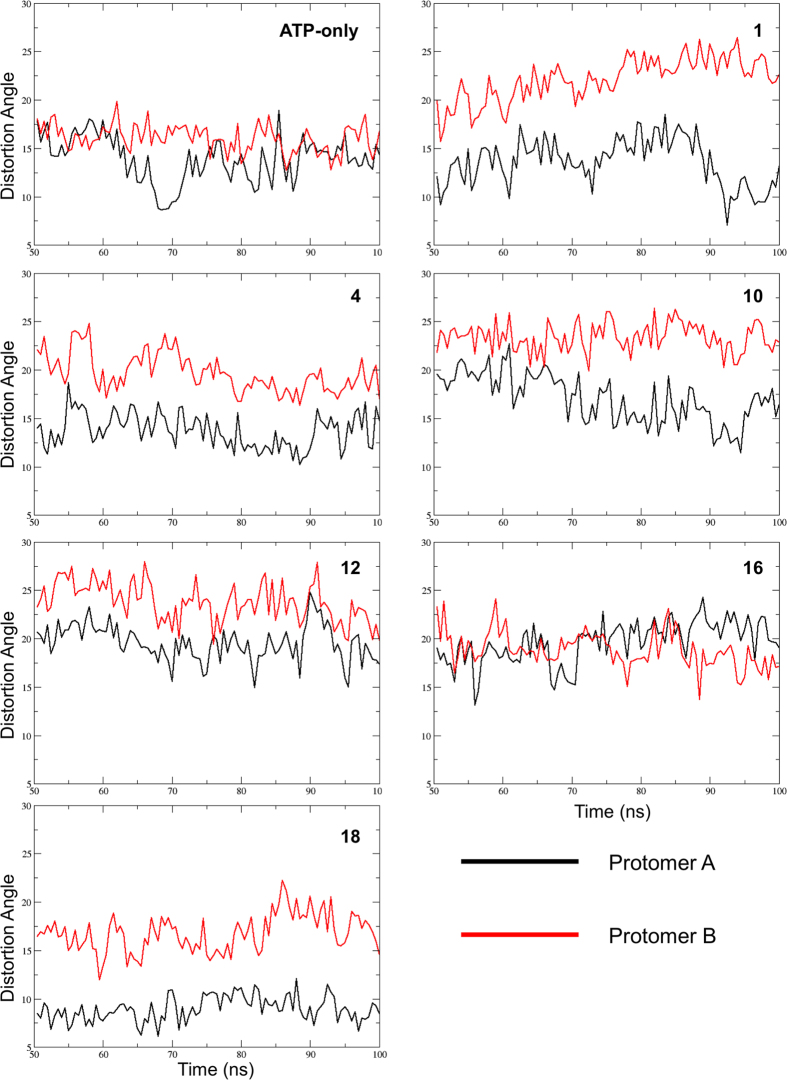
Time evolution of the distortions in the main axes of the two protomers relative to the 2CG9.pdb symmetric structure. After alignment of the single protomers belonging to snapshots from the simulations in the presence of the allosteric activators on yHsp90 structure (2CG9.pdb) significant differences between the two protomers are observed except for the cases of ATP-only and of the complex with compound 16. The black trace refers to protomer A; the red trace refers to protomer B.

**Figure 4:**
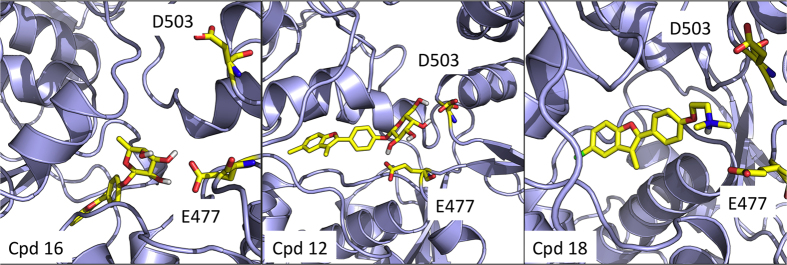
Representative structures of the complexes of Hsp90 with allosteric ligands. The figure shows the structures of the complexes of Hsp90 with the compound showing the least activation of ATPase (16), and with more potent stimulators, such as **12** and **18**. The compounds and Hsp90 residues E477 and D503 are highlighted in yellow.

In the absence of the allosteric ligands, the two protomers overall show a similar behavior with a limited distortion relative to the starting conformation. In contrast, the presence of the activators consistently determines a clear distinction between the two protomers, with protomer B, which contacts the allosteric ligand more extensively, being significantly more distorted than protomer A (Fig. 3). The only exception to this behavior is represented by the complex formed with compound **16,** whose profile is highly similar to that of the ATP-only complex. Interestingly, **16** is the derivative showing the least effect on ATPase activity (see Table 1). The rationale for this different behavior can be found in the specific structure of the carbohydrate moiety (β-L-Fucose) in **16**: the *cis* configuration of the two hydroxy groups at positions C-3 and C-4 and the lack of an OH group at position C-6 of the sugar do not favor the formation of stable hydrogen bonding interactions with the side chains of E477 and D503 on one of two protomers, which are located on opposite sides of the sugar ring. These residues can be ideally targeted by *trans* OH groups on atoms C-3 and C-4, as well as by on OH substitution on C-6, as in the case of compounds **1**, **4**, **10**, and **12** (see Fig. 4). In the case of compound **18**, the positively charged tertiary amino-group acts as a bridge between the two negatively charged side chains of E477 and D503, through electrostatic interactions. Such network of interactions induces strain in the 3D structure of the protein, which eventually results in the observed distortion in one of the two protomers, namely the one to which the two interacting E477 and D503 side chains belong. The structural details at the basis of the activity of the different ligands are also described in our previous paper on allosteric design^12^.

In this framework, the distortions induced by the activators could favor the population of the asymmetric state proposed by Agard and coworkers as a high-energy intermediate for the activation of ATP hydrolysis^28^,^29^.

### Effects of Allosteric Ligands on Hsp90 Internal dynamics

Next, we turned our attention to the characterization of the effects of allosteric ligands on the internal dynamics of Hsp90, compared to the ATP-only case. We first characterized the global rigidity/flexibility patterns in the complexes by calculating the fluctuations of pairwise amino acid distances (this analysis was also named coordination propensity (CP) analysis in previous papers^30^,^31^,^32^). To obtain a compact representation of the data for the different compounds, we calculated the difference matrix between all residue pair fluctuations determined for the ATP-only case and all residue pair fluctuations determined in the presence of the allosteric ligands. Positive values in the matrix (black color) indicate higher flexibility in the ATP-only state, while negative ones (blue color) indicate higher flexibility in the allosteric complex. This calculation is then intended to pinpoint the distribution of rigidity/flexibility blocks within the protein. As a consequence of ligand binding, amino acids belonging to different domains may change their long-distance coordination, coupled to a modulation of the inter-domain rigid dynamics.

**Figure 5:**
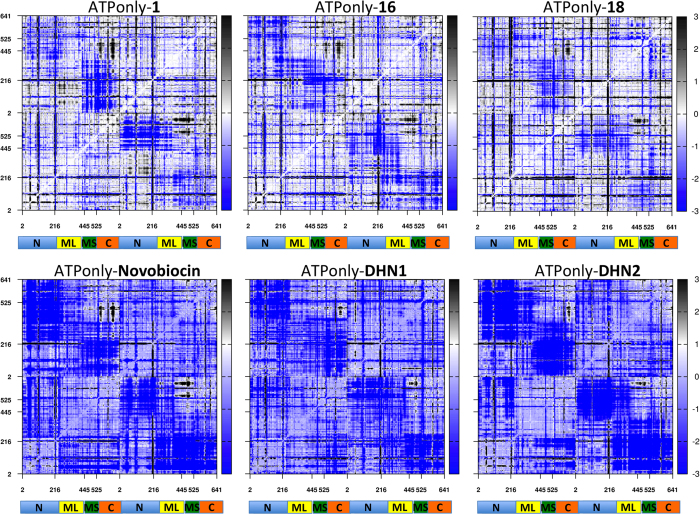
Difference distance fluctuation matrices for Hsp90 in complexes with different ligands. The matrices are calculated as the difference between the magnitude of pairwise distance fluctuations in ATP-only simulation and the respective pairwise distance fluctuation in complex with the indicated allosteric ligand. The matrices are color coded, from blue (higher flexibility in the complex with an allosteric ligand) to black (higher flexibility in the ATP-only state).

In the presence of the activators (Fig. 5 Upper Panel), the patterns of flexibility/rigidity are highly similar to the ones of the ATP-only case, as shown by the large white regions, the main differences being that in the allosteric complexes the NTDs from one protomer (represented as a light blue domain in the figure) are decoupled from the block formed by the Small portion of the Middle Domain (Small-Middle Domain, SMD) and the CTD (represented as a light red and green domains in the figure) of the other, as indicated by the larger blue-colored blocks. Interestingly, the split of the Middle domain into a Large and a Small subdomain is consistent with the domain separation observed in the X-ray structure of the mitochondrial Hsp90 homolog TRAP1 and with evolutionary studies indicating that Hsp90 and other GHKL family members significantly diverge at this subunit^33^. These results once more suggest a peculiar role for the SMD-CTD unit in regulating functionally oriented aspects of Hsp90 conformational dynamics.

By contrast, the high degree of internal coordination between the NTD and MD within each protomer was maintained upon compound addition, indicated by the presence of extended white blocks. As observed previously, intraprotomer coordination of the two domains that make up the active site favors structural preorganization for catalysis. In parallel, interprotomer flexibility can be aptly exploited to speed up the search for the closed active state, consistent with the observed increases in ATPase.

In contrast, control simulations run in the presence of coumarin-based inhibitors indicate a uniform increase in the overall protein flexibility, likely favoring distortions of both protomers and detachment of the two NTDs (Fig. 5 Lower Panel). This enhanced flexibility upon addition of a compound is quite unusual as compound binding normally stabilizes proteins, reducing dynamics. Thus, it may be a hallmark of allosteric inhibitors to work by increasing dynamics, favoring transitions away from the ground and catalytically active states. In the case of activators, the modulation of flexibility may work by facilitating Hsp90 to transition through conformations of the catalytic cycle.

### A Quantitative Structure-Dynamics-Activity Relationship Model for Designed Compounds

The design and selection of small molecule inhibitors targeting well-defined enzymatic catalytic sites is now routinely accessed through virtual screening. On the other hand, experimental screening approaches can be used to select for a certain protein activity. The Wells lab demonstrated the possibility to select for specific kinase and caspase activators by combining a tethering approach with crystal structure studies^7^,^34^. Work by the Buchner lab proved the feasibility of using high-throughput FRET based assays for the discovery of artificial activators of Hsp90 35.

However, the computational design of allosteric protein activators still represents a challenging task, in general and for Hsp90 in particular. The dynamic nature of the allosteric binding sites, combined with the fact that in many cases no direct correlations between the ligand binding affinity values and any effects on chaperone functions or cellular activity can be observed, makes the problem of structure-based activators design particularly complex^36^,^37^,^38^.

To progress along this difficult but fascinating avenue, we set out to explicitly consider the dynamic cross-talk between the chaperone and allosteric activators as an integral part of the development of a *structure-dynamics-activity relationship* (SDAR) model. In other words, we made direct use of the dynamic adaptation of protein conformations to the presence of the ligand (and *vice versa*) in deriving a simple model relating the physico-chemical properties of the small molecules to their activating effects. On this basis, we chose to describe binding in terms of an ensemble of ligand poses in complex with an ensemble of protein structures: in this way, protein and ligand conformations that could play a role in the activation process may be captured and recapitulated in a simple model.

**Figure 6:**
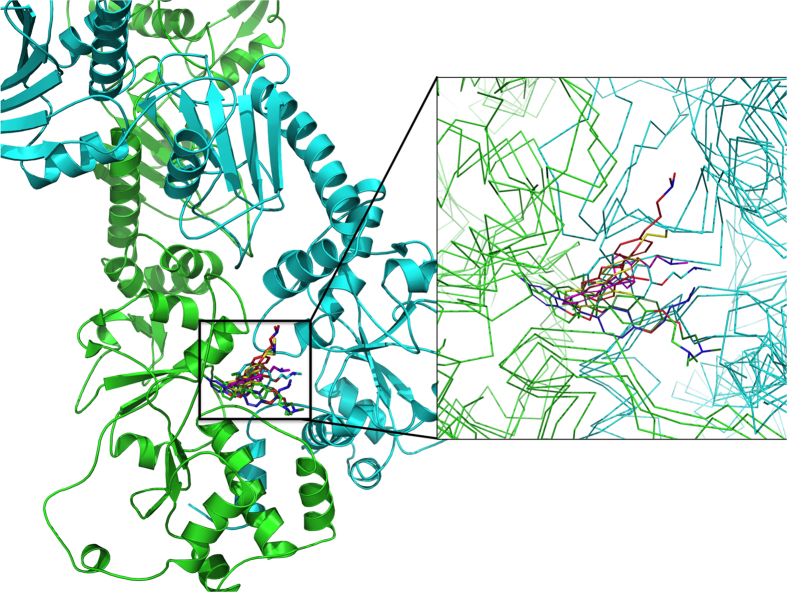
Multiple binding poses of ligand 18, adapting to multiple conformations of Hsp90. The conformational and dynamical variability of the protein in response to the ligand (and vice versa). The inset shows a zoom into the allosteric binding site.

To achieve this goal, we adopted a *Docking* over *Multiple Receptor Structures* (DMRS) approach, originating from the ATP-only dynamics: the 10 representatives of the most populated conformational clusters obtained from the ATP-only simulation were extracted from the MD trajectory and compounds **1**, **4, 10, 12, 16, 18** were re-docked into each of the 10 representative structures (see Materials and Methods) (Fig. 6 exemplifies this for compound **18**). The Ligand Efficiency (LE) for the best pose of each compound in each of the 10 target Hsp90 structures was calculated. Ligand efficiency (LE) is a measure of the average binding energy per atom in a certain ligand^39^ and allows comparison of different sized molecules binding the same target. The average LE over the best poses obtained from the DMRS calculation (Table 1 – Avg. LE on ATP-only column) strongly correlates for each activator with the experimental ATPase rate (Table 1 – ATPase), thus representing a predictive descriptor for the evaluation of new modulators. The combination of DMRS and LE computation provides a convenient common structural framework to design, dock and evaluate novel compounds, without the need for additional computationally expensive MD simulations. As a control, for each activator, we also evaluated the average LE from dynamic trajectories of the respective Hsp90 complexes, using the representatives of the most populated structural clusters for each dynamics (Table 1 - Avg. LE on Clusters) and on random snapshots extracted from the equilibrated parts of the trajectories (Table 1 - Avg. LE on Snapshots). The resulting LE values for each ligand were then averaged and correlated to the respective experimentally determined ATPase stimulations (Table 1). Interestingly, the correlation coefficients resulted to be higher (−0.90) using the docked poses in the 10 protein structures from the ATP-only simulation, than using the representative structures of the most populated conformational clusters of the complex dynamics (−0.70), or using the representative snapshots extracted from the trajectories of the same dynamics (−0.57). These data indicate that LE evaluated on multiple protein structures can aptly capture the salient determinants of observed ATPase activation profiles and allow to extract quantitative structure-dynamics-activity relationships for a series of modulators. Control calculations using only one single best pose yielded poor correlations in the order of −0.10. This result underlines the importance of using multiple poses of the allosteric ligands in contact with different conformations of the chaperone to facilitate the generation of a model that quantitatively correlates the structures and dynamics of ligand/protein complexes to experimentally observed activation of ATPase activity (Fig. 6). To the best of our knowledge, this is one of the first fully rational structure-dynamics-activity models for the modulation of chaperone functions induced by allosteric small molecule ligands.

We also evaluated the correlations between average LE values and FRET closure rates. A significant correlation of −0.76 could be obtained only when using the poses from DMRS (values of −0.40 and −0.30 were obtained for the structures obtained from the complex dynamics clusters and snapshots, respectively). We speculate that in this latter case, larger conformational changes are relevant to correctly describe the closure process and sampling limitations play a key role.

Though based on a limited number of compounds, which is a necessary condition given the high computational costs of all-atom MD simulations, our data overall support a model whereby the allosteric small molecules stabilize specific protein conformations selected from the ensemble around the closed activated state, possibly providing a path towards precise control of Hsp90 reactivity.

**Figure 7:**
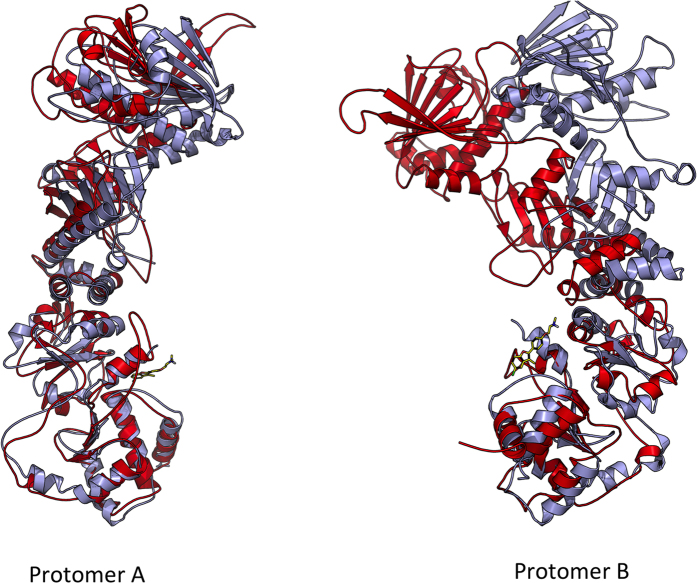
Asymmetric Distortion Induced by Compound 18. Global alignment of the protomers of Hsp90 in the ATP-only state (light blue) and in complex with ligand 18 (red) shows significant differences in the distortions of protomer A and B.

Indeed, the analysis of the effects of compounds on the structural distortions of Hsp90 highlighted relevant patterns for activation. Consistent with the characterization of the time evolution of the angle formed by the principal axes of the N-M domains of each protomer compared to the 2CG9.pdb crystal structure, an evident asymmetry in the two protomers emerges in the presence of the allosteric activators, with protomer B (the one in contact with the variable functional groups installed on the benzofuran scaffold) consistently showing a larger distortion than protomer A (Fig. 7).

In this context, it is possible to hypothesize that different ligand poses modulate the ensemble of protein conformations responsible for enzymatic activities. The coupling of dynamic ligand binding to distinct active protein conformations defines thus the mechanism for allosteric activation.

### The Cross-Talk between Protein and Allosteric Ligand Determines Allosteric Activities

The possibility of characterizing the dynamic properties of a protein’s active state can provide a rational basis for designing chemical stimulators of its function. The main challenges in this framework involve the advancement of drug design to explicitly include the characterization of protein functional dynamics, the development and experimental testing of compounds able to directly modulate the functional substates of the target through selected functional groups, and the investigation of the mechanistic consequences of allosteric binding on the functionally oriented dynamic properties of the protein. Meeting these challenges can directly contribute to advancing the role of chemical design in the investigation of complex biomolecular systems. The long-term perspective is in fact the definition of methods for the rational development of new chemical entities able to modulate and not only inhibit protein activities linked to cellular functions.

Our work demonstrates that the cross-talk between allosteric ligands and protein conformations can dynamically modulate protein activities. In the conformational selection model of binding and activity, a small molecule can preferentially target and stabilize key functional sub-states from an ensemble of conformations available on the protein free energy landscape. This concept was for instance at the basis of the design of Gleevec, a potent inhibitor of kinase activities, which binds differently to the active conformation of a kinase (Syk) than to the inactive conformation of another (Abl)^40^.

In this context, we have previously combined conformational selection concepts and studies of nucleotide-regulated dynamics for the discovery of an allosteric site in Hsp90 30,31,32. Next, we used the knowledge of the stereoelectronic properties of the allosteric site to select a small molecule lead binder. This was then evolved into a small library of derivatives showing the ability to allosterically activate Hsp90 ATPase and closure dynamics. More potent activators were then designed through the rational modification of the functionalities decorating the benzofuran scaffold to exploit potential productive interactions with the network of charged amino acids in the allosteric pocket, resulting in compounds able to accelerate Hsp90 enzymatic activity and closure kinetics by up to 6 times^12^.

Building on these results, herein we set out to generate a comprehensive structural, dynamic and activity model to link the presence of specific allosteric ligands to the experimentally observed activation of Hsp90 functional stimulation. Binding of allosteric activators determines the stabilization of the closed N-terminal dimerized state of the chaperone necessary for catalysis and function, as shown by the consistent increase in the number of NTD-NTD contacts during MD simulations. Control simulations in the presence of known inhibitors show a clear disruption of the NTD-NTD contacts.

In parallel, analysis of Hsp90 internal dynamics using the distance fluctuation Coordination Propensity (CP) parameter highlights lower interprotomer coordination in the presence of the allosteric activators. In this case, an asymmetric behavior emerges as well: in protomer A, in fact, high coordination entails all the NTD and M-large regions. The subdomains of protomer B, on the other hand, show patterns similar to those observed for the ATP-only cases.

Although never previously observed with a eukaryotic cytosolic Hsp90, the proposed formation of an asymmetric, activated state is consistent with recent results by the Agard Lab based on the crystal structures of the mitochondrial Hsp90 Trap1 and studies on the bacterial Hsp90 HtpG. These results indicate that after ATP binding at the NTD, a high-energy asymmetric state is populated which favors sequential ATP hydrolysis by the two protomers^28^,^29^. The ligand-induced increased flexibility coupled to the differential structural and dynamic profiles between the two protomers observed here can favor the global rearrangements that lead to and stabilize the formation of the high-energy asymmetric catalytically competent state, eventually resulting in ATPase stimulation.

## Conclusions

Overall, our findings establish that the interactions between the allosteric ligands and the closed active conformations of Hsp90 can direct functional dynamics towards the preferential population of compact asymmetric states, in which conformers of the chaperone that are optimally poised for ATPase are selected, collectively determining the observed biological activity. This model is consistent with the description of allosteric activation events recently proposed by Nussinov and coworkers^41^, whereby different states of a protein can be separated by a certain surmountable free energy barrier, and binding of the allosteric effectors shifts the population eliciting allosteric effects. In this context, the small molecule effector acts as a conformational catalyst that favors the protein’s transition to the active state, lowering the barrier for the population shift. Moreover, our work also highlights the functional importance of transient asymmetric states in otherwise symmetric protein assemblies.

While still hampered by the inherent limitations of MD sampling for such a large and complex system as Hsp90, our work begins to define how allosteric ligands can elicit modulated responses in their target proteins, while at the same time shedding light on the underlying mechanisms of protein dynamics and setting the stage for the use of computational approaches to rationally design chaperone activators. These aspects can have important implications in the development of allosteric modulators with graded activities for a number of relevant targets, including for instance nuclear receptors, kinases, caspases and GPCRs. In all these contexts, the availability of chemical tools that allow the control and modulation of the functional dynamics of proteins (and their networks) can have fundamental impact on physiology studies and the development of novel mechanism-based therapeutics.

## Materials and Methods

### Molecular Dynamics Simulations

The starting structures of MD simulations of Hsp90 in complex with the allosteric ligand compounds were obtained from docking calculations, using the most representative protein conformation of previous MD simulations of Hsp90 in the ATP bound state^30^. The receptor preparation was performed using the “Protein Preparation Wizard” suite of the MAESTRO program (see www.Schrodinger.com). Bond orders and atomic charges were assigned, and hydrogen atoms were added. The assignments of protonation states for basic and acidic residues were based on the optimization of hydrogen bonding patterns. The final minimization of the protein was performed with the default parameters of the Preparation Wizard. The allosteric binding site previously defined^30^ was mapped onto a grid with dimensions of 36 Å (enclosing box) and 14 Å (ligand diameter midpoint box), centered on residues 474–487, 502–503, 591–599, 602–603, and 652–657 from chain A and residues 502–504, 591–595, and 656–662 from chain B (Hsp90 residues numbering as in the PDB entry 2CG9^21^. Docking calculations were performed using the program Glide (version 5.8 Schrödinger, LLC, New York, NY, 2012)^42^,^43^, keeping the receptor rigid and allowing flexibility for the ligand. Docking calculations were performed in Standard Precision mode (SP) with the OPLS-AA (2001) force field^44^, non-planar conformations of amide bonds were penalized, Van der Waals radii were scaled by 0.80, and the partial charge cutoff was fixed to 0.15. No further modifications were applied to the default settings.

The best poses of the docking runs, according to the Emodel empirical scoring function implemented in MAESTRO (www.Schrodinger.com), of the ATP-bound state of Hsp90 together with allosteric compounds (Novobiocin derived inhibitors and Benzofuran based activators) were then used as a starting point for all-atom MD simulations in explicit water.

The simulation and the analysis of the trajectory were performed using the GROMACS^45^ software package using the GROMOS96 53A6 force field^46^. In order to remove any bad contacts, the complex was initially minimized in vacuo by multiple minimizations (200 steps steepest descent plus 200 steps conjugate gradient). Then the system was solvated in a tetrahedral box large enough to contain the protein and 1.0 nm of solvent around it on each side. The SPC water model was used for solvation^47^, and Na+ counterions were added to ensure electroneutrality. In order to allow the solvent molecules to relax around the solute, the system was minimized with position restraints on the protein and ligand and just minimizing the positions of water and ions (500 steps steepest descent plus 200 steps conjugate gradient). After this minimization step, the whole system was equilibrated by a 100 ps of MD run in the NVT ensemble to allow the temperature reaching a plateau at 300 K^48^. The first equilibration run was followed by a second 100 ps run in the NPT ensemble to adjust the pressure and the density of the system, by weak coupling to a bath of constant pressure (P0 = 1 bar, coupling time τP = 0.5 ps)^48^. The first 20 ns of the trajectory were not used in the subsequent analysis in order to minimize convergence artifacts.

Equilibration of the trajectory was checked by monitoring the equilibration of quantities, such as the root-mean-square deviation (rmsd) with respect to the initial structure, the internal protein energy, and fluctuations were calculated on different time intervals. The electrostatic term was described by using the particle mesh Ewald algorithm^49^, the LINCS algorithm^50^ was used to constrain all bond lengths and the SETTLE algorithm^51^ was used for water molecules. A dielectric permittivity, ε = 1, and a time step of 2 fs were used. All atoms were given an initial velocity obtained from a Maxwellian distribution at the desired initial temperature of 300 K. In all simulations, the temperature was maintained close to the intended values by weak coupling to an external temperature bath with a coupling constant of 0.1 ps. The proteins and the rest of the system were coupled separately to the temperature bath^48^. Each Hsp90/allosteric molecule complex was run for 100 ns. Three replicas were run for each complex.

All analyses were carried out with the tools available in the Gromacs suite and with in house developed software. For the contact analysis reported in Fig. 2, a contact is considered to be present if the distance between any pairs of atoms from two residues belonging to different protomers was lower than 6 Å.

### Protein internal dynamics

To analyze the impact of each compound on protein internal dynamics, we made use of the previously introduced distance fluctuation analysis. For each MD trajectory in complex with an allosteric ligand (activator or inhibitor) we computed on the time interval 25–100 ns (the first 25 ns trajectory are removed in order to avoid equilibration artifacts), the matrix of distance fluctuations, corresponding to the *CP* parameter^31^:


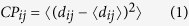


where *d*_*ij*_ is the (time-dependent) distance of the *Cα* atoms of amino acids *i* and *j* and the brackets indicate the time-average over the trajectory. Notice that *CP* is invariant under translations and rotations of the molecules and, unlike the covariance matrix, does not depend on the choice of a particular protein reference structure. The *CP* matrix, and various quantities derived from it, can be used to characterize the salient elasticity and plasticity properties of a protein undergoing structural fluctuations. The presence of specific coordination patterns and quasi-rigid domains in the protein of interest should reflect in specific properties of *CP*. In fact, pairs of amino acids belonging to the same quasi-rigid domain should be associated with much smaller distance fluctuations than amino acid pairs in different domains.

The CP values obtained from three different 100 ns MD trajectories for each allosteric ligand were averaged. The same was done with the matrix corresponding to the ATP-only case. We first characterized the global effects of ligands on chaperone dynamics by calculating the fluctuations of pairwise amino acid distances on all possible residue pairs: the data obtained for the simulations of Hsp90 in complex with both ATP *plus* allosteric ligands were subtracted with the ones obtained for the chaperone in complex with ATP *only*, and reported in the matrices analyzed in the text. This allws to identify residues that change their coordination upon ligand binding.

### Axes and angles

In order to investigate the internal rearrangement of the protein induced by the compounds, in particular the asymmetry of the protein and the distortion within each monomer, we defined two different axes of inertia of every protomer and then we determined the two angles between them. To do this, all the trajectories were sampled every 500 ps starting at 50 ns, obtaining 100 structures per simulation. Using the program Chimera (v 1.9)^52^ each monomer of all protein conformations was superimposed on the CTD of the corresponding monomer in the crystal structure of Hsp90 (PDB code: 2CG9). Two axes per monomer were defined: the first along the entire protomer and the second comprising only the NTD and Middle domains. To determine the axes, in all cases we used only residues forming the conserved secondary elements along the simulation time.

We then measured all the angles formed by NM axis of this protomer and all the other protomers, considering the average on the three replicas. Finally we calculated the difference (NM monomer B - NM monomer A) of each structure, reporting the asymmetrical rearrangement of the protein along the simulation time due to the presence of different compounds. In order to highlight the internal deformation of each protomer, we also measured the angle between the entire monomer and its NM axis considering, as stated above, the average values of the replicas and then the differences (chain B - chain A).

### Structural Cluster Analysis

Structural cluster analysis of the trajectories was carried out by the method described by Daura and coworkers^53^. Briefly: count the number of neighbors using a cut-off of 0.2 nm for RMSD between the optimal backbone superimposition of the sampled structures, then take the structure with the largest number of neighbors with all its neighbors as cluster and eliminate it from the pool of clusters. This procedure is repeated for the remaining structures in the pool. The RMSD was calculated on the backbone atoms of the C-terminal domain. We kept the first ten structures for the docking calculations, representing more than 95% of the structure variability.

### Quantitative Structure-Dynamics-Activity Relationships (QSDAR)

The first ten most populated structures resulting from the cluster analysis of the MD simulations of Hsp90 in the ATP bound state described in^30^ without any ligand at CTD were used for docking calculations in the DMRS approach. To extract control data from the complex dynamics, the representative structures of the 6 most populated clusters resulting from the combined analysis of the MD trajectories produced for the closed ATP-bound Hsp90 in complex with compounds **1, 4, 10, 12, 16, 18** (Table 1 - clusters) or the structures of 6 snapshots (for each ligand complex) extracted from the equilibrated part of the same MD trajectories (Table 1 – snapshots) were used. In DMRS, re-docking calculations were carried out using the grid described in the Molecular Dynamics Simulations paragraph. Docking calculations were carried out with the program Glide^43^ in Extra Precision mode (XP) with standard OPLS-AA (2001) force field and, in order to refine the conformational search, 20000 poses per ligand for the initial phase of docking were saved with a scoring window for keeping initial poses within 200 kcal/mol, keeping up to the best 800 poses per ligand for successive energy minimization.

In the second and third case, LE was simply calculated as a Glide score given calculated with MAESTRO on the respective structures.

At the end of calculation the experimental data of the ATP-ase activity and FRET measurements were correlated by Pearson coefficient with the Average Ligand Efficiency calculated based on the Glide-score, given by Glide in all ten conformations of the complexes (results are shown in Table 1).

## Additional Information

**How to cite this article**: Vettoretti, G. *et al.* Molecular Dynamics Simulations Reveal the Mechanisms of Allosteric Activation of Hsp90 by Designed Ligands. *Sci. Rep.* **6**, 23830; doi: 10.1038/srep23830 (2016).

## Figures and Tables

**Table 1:** Correlations between Ligand Efficiencies (LE) and Experimentally determined Hsp90 ATPase stimulation.

Allosteric Ligand	Avg. LE on ATP-only^a^	Avg. LE on Clusters^b^	Avg. LE on Snapshots^c^	ATPase (ATP turnover rate, min^−1^)^d^
1	−0.24 (0.02)	−0.24 (0.04)	−0.24 (0.04)	1.24
4	−0.25 (0.03)	−0.25 (0.03)	−0.25 (0.03)	2.27
10	−0.28 (0.05)	−0.23 (0.04)	−0.23 (0.05)	2.74
12	−0.28 (0.01)	−0.29 (0.03)	−0.28 (0.03)	2.35
16	−0.21 (0.04)	−0.23 (0.03)	−0.26 (0.03)	1.17
18	−0.30 (0.03)	−0.30 (0.03)	−0.30 (0.04)	4.01
LE–ATPase Correlation:	−0.90	−0.70	−0.57	

^a^LE of ligand averaged over the best pose value in 10 different protein structures of the ATP-only simulation (see text).

^b^LE averaged calculated on the representative structures of the 6 most populated clusters from each simulation.

^c^LE averaged calculated on 6 snapshots extracted from the trajectories of each ligand.

^d^From ref. 12.

## References

[b1] Bahar I., Chennubhotla C. & Tobi D. Intrinsic dynamics of enzymes in the unbound state and, relation to allosteric regulation. Curr. Op. Struct. Biol. 17, 633–640 (2007). 10.1016/j.sbi.2007.09.011 PMC2197162 18024008

[b2] Smock R. G. & Gierasch L. M. Sending signals dynamically. Science 324, 198–203 (2009). 19359576 10.1126/science.1169377 PMC2921701

[b3] Tsai C. J., del Sol A. & Nussinov R. Protein allostery, signal transmission and dynamics: a classification scheme of allosteric mechanisms. Mol. BioSyst. 5, 207–216 (2008). 19225609 10.1039/b819720b PMC2898650

[b4] Jorgensen W. L. Challenges for academic drug discovery. Angew. Chemie Intl. Ed. 51, 11680–11684 (2012). 10.1002/anie.201204625 23097176

[b5] Gether U. & Kobilka B. K. G Protein-coupled receptors II. Mechanism of agonist activation. J. Biol. Chem. 273, 17979–17982 (1998). 9660746 10.1074/jbc.273.29.17979

[b6] Hardy J. A. & Wells J. A. Searching for new allosteric sites in enzymes. Curr. Opin. Struct. Biol. 14, 706–715 (2004). 15582395 10.1016/j.sbi.2004.10.009

[b7] Zorn J. A. & Wells J. A. Turning enzymes ON with small molecules. Nat. Chem. Biol. 6, 179–188 (2010). 20154666 10.1038/nchembio.318

[b8] De Smet F., Christopoulos A. & Carmeliet P. Allosteric targeting of receptor tyrosine kinases. Nat. Biotech. 32, 1113–1120 (2014). 10.1038/nbt.3028 25380447

[b9] Nussinov R. & Tsai C.-J. Allostery in disease and drug discovery. Cell 153, 293–305 (2013). 23582321 10.1016/j.cell.2013.03.034

[b10] Panjkovich A. & Daura X. Exploiting protein flexibility to predict the location of allosteric sites. BMC Bioinformatics 13, 273 (2012). 23095452 10.1186/1471-2105-13-273 PMC3562710

[b11] del Sol A., Tsai C.-J., Ma B. & Nussinov R. The origin of allosteric functional modulation: multiple pre-existing pathways. Structure 17, 1042–1050 (2009). 19679084 10.1016/j.str.2009.06.008 PMC2749652

[b12] Sattin S. *et al.* Activation of Hsp90 Enzymatic Activity and Conformational Dynamics through Rationally Designed Allosteric Ligands. Chemistry 21, 13598–13608 (2015). 26286886 10.1002/chem.201502211 PMC5921052

[b13] Taipale M., Jarosz D. F. & Lindquist S. Hsp90 at the hub of protein homeostasis: emerging mechanistic insights. Nat. Rev. Mol. Cell Biol. 11, 515–528 (2010). 20531426 10.1038/nrm2918

[b14] Echeverria P. C., Bernthaler A., Dupuis P., Mayer B. & Picard D. An Interaction Network Predicted from Public Data as a Discovery Tool: Application to the Hsp90 Molecular Chaperone Machine. Plos One 6, e26044 (2012). 22022502 10.1371/journal.pone.0026044 PMC3195953

[b15] Kirschke E., Goswami D., Southworth D., Griffin P. R. & Agard D. A. Glucocorticoid receptor function regulated by coordinated action of the Hsp90 and Hsp70 chaperone cycles. Cell 157, 1685–1697 (2014). 24949977 10.1016/j.cell.2014.04.038 PMC4087167

[b16] Taipale M. *et al.* Quantitative analysis of Hsp90-client interactions reveals principles of substrate recognition. Cell 150, 987–1001 (2012). 22939624 10.1016/j.cell.2012.06.047 PMC3894786

[b17] Whitesell L. & Lindquist S. L. Hsp90 and the chaperoning of cancer. Nat. Rev. Cancer 5, 761–772 (2005). 16175177 10.1038/nrc1716

[b18] Shah V. *et al.* Hsp90 regulation of endothelial nitric oxide synthase contributes to vascular control in portal hypertension. Am. J. Physiol. 277, G463–468 (1999). 10444461 10.1152/ajpgi.1999.277.2.G463

[b19] Luo W., Sun W., Taldone T., Rodina A. & Chiosis G. Heat shock protein 90 in neurodegenerative diseases. Mol. Neurodegener 5, 24 (2010). 20525284 10.1186/1750-1326-5-24 PMC2896944

[b20] Krukenberg K. A., Street T. O., Lavery L. A. & Agard D. A. Conformational dynamics of the molecular chaperone Hsp90. Q. Rev. Biophys. 44, 229–255 (2011). 21414251 10.1017/S0033583510000314 PMC5070531

[b21] Ali M. M. U. *et al.* Crystal structure of an Hsp90-nucleotide-p23/Sba1 closed chaperone complex. Nature 440, 1013–1017 (2006). 16625188 10.1038/nature04716 PMC5703407

[b22] Shiau A. K., Harris S. F., Southworth D. R. & Agard D. A. Structural analysis of E-coli Hsp90 reveals dramatic nucleotide-dependent conformational rearrangements. Cell 127, 329–340 (2006). 17055434 10.1016/j.cell.2006.09.027

[b23] Dollins D. E., Warren J. J., Immormino R. M. & Gewirth D. T. Structures of GRP94-Nucleotide complexes reveal mechanistic differences between the Hsp90 chaperones. Mol. Cell. 28, 41–56 (2007). 17936703 10.1016/j.molcel.2007.08.024 PMC2094010

[b24] Ratzke C., Mickler M., Hellenkamp B., Buchner J. & Hugel T. Dynamics of heat shock protein 90 C-terminal dimerization is an important part of its conformational cycle. Proc. Natl. Acad. Sci. USA 107, 16101–16106 (2010). 20736353 10.1073/pnas.1000916107 PMC2941327

[b25] Mickler M., Hessling M., Ratzke C., Buchner J. & Hugel T. The large conformational changes of Hsp90 are only weakly coupled to ATP hydrolysis. Nat. Struct. Mol. Biol. 16, 281–286 (2009). 19234469 10.1038/nsmb.1557

[b26] Genest O. *et al.* Uncovering a region of heat shock protein 90 important for client binding in E. coli and chaperone function in yeast. Mol. Cell 49, 464–473 (2013). 23260660 10.1016/j.molcel.2012.11.017 PMC3570620

[b27] Street T. O. *et al.* Elucidating the mechanism of substrate recognition by the bacterial Hsp90 molecular chaperone. J. Mol. Biol. 426, 2393–2404 (2014). 24726919 10.1016/j.jmb.2014.04.001 PMC5322795

[b28] Lavery L. A. *et al.* Structural asimmetry in the closed state of mitochondrial Hsp90 (TRAP1) supports a two-step ATP hydrolysis mechanism. Mol. Cell. 53, 330–343 (2014). 24462206 10.1016/j.molcel.2013.12.023 PMC3947485

[b29] Partridge J. R. *et al.* A novel N-terminal extension in mitochondrial TRAP1 serves as a thermal regulator of chaperone activity. eLife, 10.7554/eLife.03487 (2014). PMC4381864 25531069

[b30] Morra G. *et al.* Dynamics-Based Discovery of Allosteric Inhibitors: Selection of New Ligands for the C-terminal Domain of Hsp90. J. Chem. Theory Comput. 6, 2978–2989 (2010). 26616092 10.1021/ct100334n PMC7575213

[b31] Morra G., Potestio R., Micheletti C. & Colombo G. Corresponding Functional Dynamics across the Hsp90 Chaperone Family: Insights from a Multiscale Analysis of MD Simulations. Plos Comput. Biol. 8, e1002433 (2012). 22457611 10.1371/journal.pcbi.1002433 PMC3310708

[b32] Morra G., Verkhivker G. M. & Colombo G. Modeling signal propagation mechanisms and ligand-based conformational dynamics of the Hsp90 molecular chaperone full length dimer. Plos Comp. Biol. 5, e1000323 (2009). 10.1371/journal.pcbi.1000323 PMC2649446 19300478

[b33] Meyer P. *et al.* Structural and functional analysis of the middle segment of Hsp90: implications for ATP hydrolysis and client protein and cochaperone interactions. Mol. Cell. 11, 647–658 (2003). 12667448 10.1016/s1097-2765(03)00065-0

[b34] Hardy J. A., Lam J., Nguyen J. T., O’Brien T. & Wells J. Discovery of an allosteric site in caspases. Proc. Natl. Acad. Sci. USA 101, 12461–12466 (2004). 15314233 10.1073/pnas.0404781101 PMC514654

[b35] Zierer B. K. *et al.* Artificial Accelerators of the Molecular Chaperone Hsp90 Facilitate Rate-Limiting Conformational Transitions. Angew. Chemie Intl. Ed. 53, 1–7 (2014). 10.1002/anie.201406578 25244159

[b36] Gestwicki J. E., Crabtree G. R. & Graef I. A. Harnessing chaperones to generate small-molecule inhibitors of amyloid beta aggregation. Science 306, 865–869 (2004). 15514157 10.1126/science.1101262

[b37] Li X. *et al.* Analogs of the Allosteric Heat Shock Protein 70 (Hsp70) Inhibitor, MKT-077, as Anti-Cancer Agents. ACS Med. Chem. Lett. 4, 1042–1047 (2013). 10.1021/ml400204n PMC3845967 24312699

[b38] Miyata Y. *et al.* Synthesis and initial evaluation of YM-08, a blood-brain barrier permeable derivative of the heat shock protein 70 (Hsp70) inhibitor MKT-077, which reduces tau levels. ACS Chem. Neurosci. 4, 930–939 (2013). 23472668 10.1021/cn300210g PMC3689201

[b39] Kuntz I. D., Chen K., Sharp K. A. & Kollman P. A. The maximal affinity of ligands. Proc. Natl Acad. Sci. 96, 9997–10002 (1999). 10468550 10.1073/pnas.96.18.9997 PMC17830

[b40] Druker B. J. STI571 (Gleevec™) as a paradigm for cancer therapy. Trends in Mol. Med. 8, S14–S18 (2002). 11927282 10.1016/s1471-4914(02)02305-5

[b41] Nussinov R., Tsai C.-J. & Li J. Principles of Allosteric Interactions in Cell Signaling. J. Am. Chem. Soc. 136, 17692–17701 (2014). 25474128 10.1021/ja510028c PMC4291754

[b42] Friesner R. A. *et al.* Glide: A new approach for rapid, accurate docking and scoring. 1. Method and assessment of docking accuracy. J. Med. Chem. 47, 1739–1749 (2004). 15027865 10.1021/jm0306430

[b43] Friesner R. A. *et al.* Extra precision glide: Docking and scoring incorporating a model of hydrophobic enclosure for protein-ligand complexes. J. Med. Chem. 49, 6177–6196 (2006). 17034125 10.1021/jm051256o

[b44] Kaminski G., Friesner R. A., Tirado-Rives J. & Jorgensen W. L. Evaluation and Reparametrization of the OPLS-AA Force Field for Proteins via Comparison with Accurate Quantum Chemical Calculations on Peptides. J. Phys. Chem. B 105 6474–6487 (2001).

[b45] Hess B., Kutzner C., van der Spoel D. & Lindahl E. GROMACS 4: Algorithms for highly efficient, load-balanced, and scalable molecular simulation. J. Chem. Theory Comput. 4, 435–447 (2008). 26620784 10.1021/ct700301q

[b46] Oostenbrink C., Villa A., Mark A. E. & van Gunsteren W. F. A biomolecular force field based on the free enthalpy of hydration and solvation: the GROMOS force-field parameter sets 53A5 and 53A6. J. Comput. Chem. 25 (2004). 10.1002/jcc.20090 15264259

[b47] Berendsen H. J. C., Grigera J. R. & Straatsma P. R. The missing term in effective pair potentials. J. Phys. Chem. 91, 6269–6271 (1987).

[b48] Berendsen H. J. C., Postma J. P. M., van Gunsteren W. F., Di Nola A. & Haak J. R. Molecular dynamics with coupling to an external bath. J. Chem. Phys. 81, 3684–3690 (1984).

[b49] Darden T., York D. & Pedersen L. Particle mesh Ewald: An N-log(N) method for Ewald sums in large systems. J. Chem. Phys. 98 (1993).

[b50] Feenstra K. A., Hess B. & Berendsen H. J. C. Improving Efficiency of Large Time-scale Molecular Dynamics Simulations of Hydrogen-rich Systems. J. Comp. Chem. 20, 786–798 (1999). 10.1002/(SICI)1096-987X(199906)20:8<786::AID-JCC5>3.0.CO;2-B 35619462

[b51] Miyamoto S. & Kollman P. A. SETTLE: An analytical version of the SHAKE and RATTLE algorithms for rigid water models. J. Comp. Chem. 13, 952–962 (1992).

[b52] Pettersen E. F. *et al.* UCSF Chimera–a visualization system for exploratory research and analysis. J. Comput. Chem. 25, 1605–1612 (2004). 15264254 10.1002/jcc.20084

[b53] Daura X. *et al.* Peptide folding: when simulation meets experiment. Angew. Chemie Intl. Ed. 38, 236–240 (1999).

